# Dentistry in the Digital Age: Embracing Blockchain Technology

**DOI:** 10.7759/cureus.39710

**Published:** 2023-05-30

**Authors:** Vineet Sharma, Kamal K Meena

**Affiliations:** 1 Prosthodontics, Rajasthan University of Health Sciences (RUHS) College of Dental Sciences, Jaipur, IND

**Keywords:** patient empowerment, interoperability, data security, blockchain technology, dentistry

## Abstract

Blockchain technology can revolutionize the dental industry by offering secure and efficient data management and patient care solutions. This editorial explores how blockchain can benefit dentistry, including enhancing data security and privacy, streamlining interoperability, facilitating supply chain management, and empowering patients. Blockchain ensures tamper-proof and authorized access to patient data, enabling dentists and patients to have peace of mind regarding data security. Interoperability between dental systems can be improved through blockchain, allowing seamless data exchange and care coordination. By leveraging the transparency and immutability of blockchain, supply chain management in dentistry can be enhanced to ensure the authenticity of dental products. Moreover, blockchain empowers patients by granting them control over their healthcare data, promoting transparency and trust in the dental industry. However, challenges such as scalability, energy consumption, regulatory compliance, and data governance need to be addressed for successful implementation. Collaboration among stakeholders, education, and the development of regulatory frameworks are crucial for maximizing the benefits of blockchain technology in dentistry. By embracing blockchain, the dental industry can create a future where patient care is secure, efficient, and patient-centric.

## Editorial

Blockchain technology is a decentralized and distributed ledger system that enables the secure recording, storing, and verifying of transactions across multiple computers or nodes. But what makes it unique is that it's decentralized, meaning there's no single authority in charge. Instead, many computers worldwide work together to maintain and update the ledger. Whenever a transaction happens, it gets added to a "block" along with other transactions. Each block is linked to the previous one, forming a chain of blocks, hence the name "blockchain." Once a block is added, it cannot be changed, making the information stored in the blockchain secure and tamper-proof. Because everyone in the network has a copy of the blockchain, they can verify and validate transactions together. This creates trust among participants, as any fraudulent activity would require changing multiple copies of the blockchain simultaneously, which is incredibly difficult. In a nutshell, blockchain is a transparent, secure, and decentralized system that allows multiple computers to work together to maintain a reliable record of transactions. It offers increased trust, security, and efficiency in various areas of our digital lives.

Blockchain technology has emerged as a transformative force across various industries, disrupting traditional systems and processes. Although commonly associated with cryptocurrencies, blockchain offers potential applications that extend far beyond financial transactions. One industry that stands to benefit greatly from blockchain adoption is dentistry. By providing a secure, transparent, and efficient solution for data management and patient care, blockchain has the power to reshape dentistry for the better. In this editorial, we will delve into how blockchain can enhance data security and privacy, streamline interoperability, facilitate supply chain management, and empower patients in the dental industry.

Enhancing data security and privacy

In the dental field, accurate and secure data management is of paramount importance. Patient records, treatment plans, and medical histories must be protected from cyber-attacks, data breaches, and unauthorized access. Unfortunately, traditional centralized data systems are vulnerable to such threats. Here, blockchain technology can offer a paradigm shift in data security. By utilizing a decentralized network and robust encryption protocols, blockchain ensures that patient data remains tamper-proof and accessible only to authorized individuals. This immutable ledger provides dentists and patients with the peace of mind that their sensitive information is protected from unauthorized modifications or breaches [1].

Streamlining interoperability

Interoperability has long been a challenge in the healthcare industry, and dentistry is no exception. Different dental systems often struggle to communicate and exchange data seamlessly. Blockchain has the potential to bridge this gap and enable frictionless data exchange between various dental practices, laboratories, and insurance providers. Smart contracts, which are self-executing contracts with predefined conditions, can automate the verification of insurance claims, eliminating manual paperwork and reducing administrative burden. By securely sharing patient records and treatment information, blockchain can improve care coordination and treatment outcomes [2].

Facilitating supply chain management

Efficient supply chain management is crucial for dental clinics as it ensures the availability of high-quality materials and products. However, the current supply chain processes in dentistry are susceptible to counterfeit products, inefficient tracking, and a lack of transparency. Blockchain technology provides a transparent and immutable ledger that can record the journey of dental supplies from manufacturers to end-users. By integrating Internet of Things (IoT) devices and sensors, it becomes possible to track and verify the authenticity of dental products in real time. This ensures that patients receive safe and reliable dental materials, contributing to their overall oral health [3].

Empowering patient ownership and consent

With greater ownership and control over their health information, patients can actively participate in their treatment decisions, leading to more personalized and effective dental care. Blockchain technology can provide additional benefits and enable new possibilities for patient participation in dental care. With blockchain, patients can have more control over their medical data. They can securely store their health information on the blockchain and grant access to healthcare providers when needed. Blockchain's cryptographic techniques ensure the privacy and security of patient data. By leveraging encryption and decentralized storage, blockchain technology can protect sensitive information from unauthorized access and potential breaches. Blockchain can facilitate sharing of treatment plans and medical records among healthcare providers involved in patient care. This allows for better coordination, collaboration, and decision-making between dentists and other medical professionals involved in the patient's treatment.

Patients can grant and revoke consent for specific treatments, procedures, or data sharing, and these permissions can be recorded on the blockchain. This ensures that treatment decisions are made with informed and active patient involvement. Blockchain technology can enable patients to provide feedback and reviews on their dental experiences, which can be recorded on the blockchain. This feedback can improve the quality of care, facilitate informed decision-making for other patients, and foster a more patient-centric approach to dentistry. Blockchain technology also holds the potential for patients to access and manage their dental insurance information seamlessly, simplifying administrative processes and improving the overall patient experience [4].

Overcoming challenges and embracing the future

While blockchain technology offers promising solutions for dentistry, it is essential to recognize the challenges and potential drawbacks associated with its implementation. Below are the key considerations that need to be addressed to maximize the benefits of blockchain while mitigating its disadvantages.

Scalability

Blockchain networks can face scalability issues when processing large transactions. As the number of participants and data increases, the network can experience slower transaction times and increased costs. This scalability challenge must be addressed for blockchain to be effectively implemented in dentistry on a larger scale.

Energy Consumption

Blockchain networks, particularly those using proof-of-work consensus algorithms like Bitcoin, require significant computational power and energy consumption. The energy-intensive nature of blockchain technology raises concerns about its environmental impact and sustainability. As the dental industry strives for eco-friendly practices, balancing the benefits of blockchain with its energy consumption is crucial.

Regulatory Considerations

The adoption of blockchain in dentistry must navigate regulatory and legal frameworks. Data privacy and security regulations, such as the General Data Protection Regulation, present challenges for storing and sharing patient data on a blockchain. Ensuring compliance with these regulations while maintaining blockchain's transparency and security benefits requires careful consideration and legal guidance.

Technical Expertise

Blockchain technology is still relatively new and complex. Its successful implementation in dentistry requires dental professionals and staff to understand the technology and its applications. Training and education programs are necessary to equip the dental workforce with the knowledge and skills needed to leverage blockchain solutions effectively.

Data Governance

Blockchain's distributed nature and immutability can create challenges regarding data governance and liability. Correcting or updating the information can be difficult if incorrect or incomplete data is recorded on the blockchain. The liability issue arises when errors occur in patient records or treatment information.

Interoperability With Legacy Systems

Integrating existing dental systems and electronic health record platforms can be challenging. Blockchain solutions must seamlessly interface with legacy systems to enable efficient data exchange and interoperability.

Collaborative efforts among industry stakeholders, technology providers, and policymakers are essential to foster innovation and create interoperable blockchain solutions specific to the dental field. By carefully navigating these challenges, the dental industry can unlock the full potential of blockchain technology to enhance patient care and revolutionize dental practices [5].

In conclusion, implementing blockchain technology in the dental industry offers a promising opportunity for enhancing data security, streamlining interoperability, improving supply chain management, and empowering patients. By leveraging a decentralized network and robust encryption protocols, blockchain ensures the protection and authorized access of sensitive patient information. Seamless data exchange between dental systems, automated insurance claim verification, and real-time tracking of dental supplies can significantly improve care coordination and product authenticity. Furthermore, blockchain empowers patients by granting them control over their healthcare data and simplifying administrative processes. While challenges exist, collaborative efforts and regulatory frameworks can pave the way for a secure, efficient, and patient-centric future in dentistry.
